# Plate fixation or intramedullary fixation of humeral shaft fractures: An updated meta-analysis

**DOI:** 10.3109/17453671003635884

**Published:** 2010-04-06

**Authors:** David J Heineman, Rudolf W Poolman, Sean E Nork, Kees-Jan Ponsen, Mohit Bhandari

**Affiliations:** ^1^Department of Orthopaedic Surgery, Onze Lieve Vrouwe Gasthuis, Amsterdamthe Netherlands; ^2^Department of Orthopaedic Surgery, Harborview Medical Center, University of Washington, Seattle, WAthe Netherlands; ^3^Trauma Unit, Department of Surgery, AMC, Amsterdamthe Netherlands; ^4^Division of Orthopaedic Surgery, McMaster University, Hamilton, ONCanada

## Abstract

**Background** The optimal approach to operative treatment of humeral shaft fractures remains debatable. Previously published trials have been limited in size and have been inconclusive regarding important patient outcome variables following treatment with either intramedullary nails or plates. We conducted a meta-analysis of available trials comparing treatment of humeral shaft fractures.

**Methods** We performed a literature search from 1967 to November 2007 in the main medical search engines and selected 4 randomized trials that compared nails and plates in patients with humeral shaft fractures and that reported on complications due to surgery. We statistically pooled patient data using standard meta-analytic approaches. Our primary outcome was the total complication rate, comprised of all complications listed in the articles included. Secondary outcomes included non-union, infection, nerve palsy, and reoperation rate. Methodology was assessed using the CLEAR NPT.

**Results** When pooling the data of the 4 trials (n = 203 patients), we did not find a statistically significant difference between implants in the rate of total complications, non-union, infection, nerve-palsy, or the need for reoperation. The studies included were small and had methodological limitations.

**Conclusions** Our meta-analysis suggests stastistically insignificant differences between plates and nails in the treatment of humeral shaft fractures. Small sample sizes, study heterogeneity, and methodological limitations argue strongly for a definitive, large trial. We recommend that this trial should be a randomized controlled trial with appropriate allocation of patients and blinding of patients and care providers and outcome assessors, and that it should include patient-important outcomes.

## Introduction

Most diaphyseal humeral fractures can be managed nonoperatively and good outcomes can be expected in most cases (Sarmiento and Latta 1999, Fjalestad et al. 2000, Sarmiento and Latta 2007). However, operative treatment is indicated under a number of circumstances including open fractures, associated neurovascular injury, proximal and distal articular extension of the fracture, and in patients with other multiple injuries (Pollock et al. 1981, Bell et al. 1985, Hegelmaier and von Aprath 1993, Brug et al. 1994, Sarmiento et al. 2002). Commonly, surgery is indicated if nonoperative management has failed to maintain the humerus in an adequately reduced position or if increasing fracture stability and comfort are not observed with time (Bennett 1936, Ring et al. 2000, Kesemenli et al. 2002, Pullen et al. 2003, Rutgers and Ring 2006, Ring et al. 2007). Operative treatment may also be indicated in cases of delayed union, nonunion, or malunion following nonoperative management. Surgical stabilization can be accomplished with different implants and techniques; the most common are open reduction with plate fixation or stabilization with intramedullary nails (Modabber and Jupiter 1998, Osman et al. 1998, Cole and Wijdicks 2007). Both techniques have certain mechanical and anatomical advantages and disadvantages. Plate fixation has the advantages of stable fixation, direct visualization, protection of the radial nerve, and sparing of the adjacent shoulder and elbow joint from injury. Intramedullary nails have the advantage of closed insertion techniques, intact periosteal blood supply, and load-sharing mechanical properties. In the literature it is proposed that proximal and distal intraarticular extension of a diaphyseal humeral fracture should be treated with plate and screw fixation—as well as fractures associated with vascular or nerve injury, floating elbow or shoulder, open fractures, and humeral non-union (combined with bone grafting).

A previous meta-analysis demonstrated a significant reduction in reoperations favoring plating over nailing in humeral shaft fractures (Bhandari et al. 2006). In this meta-analysis, a risk reduction of 74% was reported for reoperations when using plates. One of the remarks made by the authors was that a larger trial was needed to be conclusive about these results. A recent randomized controlled trial comparing intramedullary nails and compression plates has suggested less favorable results with plating (Changulani et al. 2007). Because of this new evidence, we decided to do an updated meta-analysis to explore whether the current literature is conclusive about the best treatment for humeral shaft fractures. Thus, the purpose of this meta-analysis was to update the review of the complete body of trials comparing plates and intramedullary nails in the treatment of humeral shaft fractures to determine the best evidence currently available.

## Methods

This systematic review and meta-analysis follows the QUOROM statement guidelines (Moher et al. 1999).

### Literature search

We performed a MEDLINE search of the literature from 1967 to November 2007, identifying the population, the intervention, and the methodology. In the PubMed database Mesh terms (“Humerus”[Mesh] OR “Humeral Fractures”[Mesh]) AND (“Bone Nails”[Mesh] OR “Fracture Fixation, Intramedullary”[Mesh] AND “Bone Plates”[Mesh]) and type of clinical trial (Randomized controlled trials and Clinical trials) were first used. A secondary free search was then performed using multiple key words (e.g. humerus OR humeral AND fracture* AND (Intramedul* OR nail*) AND plate*) to ensure inclusion of all possible studies. Additional searches using the Embase, Cochrane, Sumsearch, Bandolier, and Trip databases were conducted using the same search terms. The database of the Orthopaedic Trauma Association's Annual Meeting Archived Presentations was searched manually for any abstracts that might be useful. Finally, we performed a Google search to identify any potentially missing articles. We did not use language as an exclusion or inclusion criterion.

Two of us (RP and DH) reviewed possible abstracts and retrieved the full article if the screening criteria were met.

### Selection criteria and study characteristics

We identified articles that met the following criteria, following the methodology of Bhandari et al. (2006):

1. Target population: individuals with fractures of the humeral diaphysis.
2. Intervention: plate fixation and intramedullary fixation.
3. Primary outcome measure: complications due to surgery (any complication following surgery; for example, nerve injury, infection, or non-union).
4. Methodology: published or unpublished, prospective and randomized or quasi-randomized controlled trials.

### Validity assessment

We evaluated all the studies included for the 4 main areas of bias. The articles were first scrutinized for evidence of selection bias, defined as bias caused by unblinded allocation to comparison groups. This included inspection for allocation concealment. Performance bias, defined as the unequal provision of care in the comparison of groups other than the intervention under investigation, was next investigated. We next evaluated all studies for detection bias, defined as prejudiced assessment of outcome. Finally, we looked for attrition bias, defined as biased occurrence of events and characterized by improper handling of deviations from protocol and loss to follow-up. All of these potential causes of bias are listed in the results section. All studies that were not randomized or quasi-randomized were considered ineligible and were not included. The checklist to evaluate a report of a non-pharmacological trial (CLEAR NPT) was used to assess these areas of bias and to describe the methodological quality of the studies that were included. This is a tool that was developed to critically appraise medical literature, to design non-pharmacological trial studies, and to assess the quality of trial reports in systematic reviews (Boutron et al. 2005). Furthermore, funnel plots were calculated to assess potential publication bias. A funnel plot is a visual aid to detect bias or systematic heterogeneity, by plotting treatment effect against a measure of study size. An asymmetric funnel plot suggests a relationship between study size and treatment effect (Alderson and Green 2002a).

### Data abstraction

All relevant data regarding patient demographics, injury characteristics, intervention, outcomes, and complications were abstracted by 2 reviewers (KJP and DH). The primary outcome measure was the difference in relative risk of the total complication rate between intramedullary nailing and plating in diaphyseal humeral shaft fractures. The complication rate was the sum of all reported complications in the articles reviewed (non-union, infection, implant failure, nerve damage, malunion, reduced ROM of the shoulder, reduced ROM of the elbow, intraoperative comminution, wound hematoma, delayed union, hardware requiring removal, impingement, shoulder pain, and elbow pain). Secondary outcomes were specified complications and included infection, non-union, nerve palsy, and reoperation rate. Nerve palsy was defined as a postoperative radial and/or posterior interosseus nerve injury.

### Quantitative data synthesis

The difference in risk ratios of the complications was then calculated. The data were pooled across studies and was used to calculate relative risks with associated 95% confidence intervals. Data were analyzed using the ReviewManager software, provided by the Cochrane Collaboration (version 4.2.10 and version 5.0.17). This software creates forest plots as a graphical representation of different treatment effects. The lines in the forest plot represent the confidence intervals and the dots represent the measures of effect. This is done for all studies separately and for all studies together, so we can see the combined treatment effect of a larger population than from one study. A random effects model was chosen to analyze the results of the different studies. In the random effects model, it is assumed that the studies used represent a sample of the available trials.

**Table:** Study characteristics

Study	Methods **^a^**	Participants	Interventions	Outcomes
Bolano et al. 1995 (abstract)	RCT	28, gender unclear	Open reduction internal fixation with DCP or rigid IMN	non-union, shoulder ROM, implant removal
Chapman 2000	RCT	84, 51 male, 33 female, mean age DCP 34 and IMN 33	Open reduction internal fixation with DCP or rigid IMN	infection, malunion, non-union, nerve injury, elbow and shoulder pain, elbow and shoulder ROM, hardware requiring removal
McCormack 2000	RCT	44, 28 male, 16 female, mean age DCP 49 age IMN 40	Open reduction internal fixation with DCP or rigid IMN	infection, non-union, implant failure, nerve injury, impingement
Changulani 2007	RCT	47, 39 male, 8 female, mean age DCP 35 and IMN 39	Open reduction internal fixation with DCP or rigid IMN	infection, non-union, implant failure, nerve injury
**^a^**RCT: Randomized controlled trial				

To assess heterogeneity, we used the chi-squared test and the “degree of freedom” value. When chi-squared is greater than the degree of freedom, there is evidence of heterogeneity (Alderson and Green 2002b). The I^2^ statistic value was calculated as a measure for determining the inter-study variability. I^2^ is useful for determining the heterogeneity due to true differences, and substantial heterogeneity was defined by an I^2^ value greater than 40% (Higgins and Thompson 2002). We hypothesized that heterogeneity, if present, was due to clinical and methodological diversity in the studies, such as study design, execution, and randomization. Accordingly, we conducted sensitivity analyses to explore any statistical heterogeneity.

Because of the substantial difference perceived in the design and function of flexible intramedullary nails (e.g. multiple-stacked, small-diameter metal implants) and rigid nails (e.g. unreamed or reamed insertion of a single medullary implant), we only used rigid nails in the primary analysis.

## Results

### Literature search and trial flow

**Figure 1.**
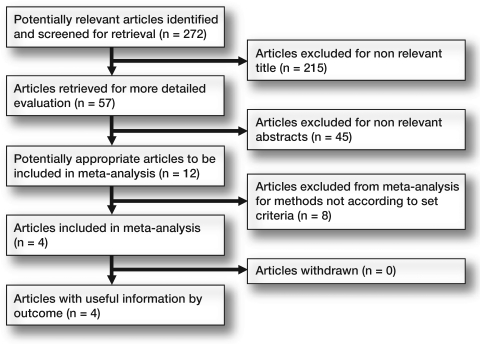
Flow chart

We identified 272 potential citations: 257 were identified in the PubMed database, 11 were found in the Embase database, and 6 were obtained from the Cochrane database. Hand-searching of the OTA meeting database resulted in 1 additional abstract. There were 57 abstracts which met the criteria for initial review. This resulted in 10 studies that underwent full-text review to investigate the methodology. Of these 10, 4 matched the inclusion criteria and were selected for this meta-analysis (Figure 1). All studies but 1 had been published in peer-reviewed journals. The search was performed in November 2007.

### Types of nails

All 4 studies reported results after the use of rigid intramedullary nails; 2 of these reported on antegrade rigid nailing (Chapman et al. 2000, Changulani et al. 2007) and 1 study included retrograde and antegrade rigid nails (McCormack et al. 2000). 1 study did not report on the method of inserting the nails (Bolano et al. 1995).

### Study characteristics

The Table describes the studies that were used in this meta-analysis and their outcomes. One study was only available as an abstract (Bolano et al. 1995). Despite several attempts, we were unable to obtain the unpublished full-text article that correponded to the published article. Since the abstract did provide sufficient details about complications in both groups, we preferred to conduct analyses with and without this paper (sensitivity analyses) rather than to exclude it altogether. The total number of patients included in all 4 studies was 203, although 6 of these patients were lost to follow-up and their data were not available. All reports described adult patients with acute humeral shaft fractures treated with plate or nail fixation. We made a table that consisted of the CLEAR NPT checklist, which describes all areas of bias. When assessing the CLEAR NPT table it was clear that the quality of the available studies was poor in general. The main areas of bias were clinical bias (antegrade versus retrograde nailing in 1 study group) and methodological bias (no blinding of patients and care providers/outcome assessors). Funnel plots were constructed to assess publication bias. These plots were widely scattered, showed asymmetry, and showed low precision of estimation of the treatment effect in the studies.

### Quantitative data synthesis

**Figure 2.**
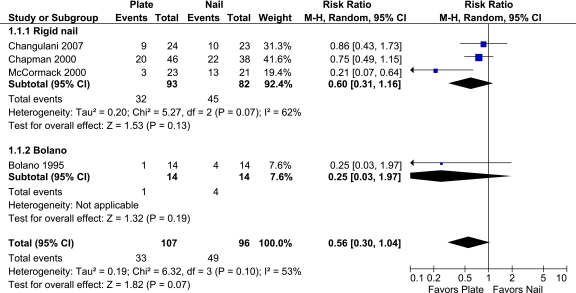
Forest plot for primary outcome: total complication rate.

*Primary outcome.* We included 203 patients from 4 studies to calculate the difference in total complication rate between the different treatment methods. The complication rate was the sum of all reported complications in the articles reviewed. The relative risk of a complication was not statistically significantly different between plate fixation and intramedullary rigid nails (RR = 0.56, 95% CI: 0.30–1.04; p = 0.07) (Figure 2). When we conducted a sensitivity analysis without the Bolano abstract, these results changed only slightly (RR = 0.60, 95% CI: 0.31–1.2; p = 0.1), but still remained statistically insignificant.

*Secondary outcomes.* The secondary outcomes of non-union, infection, nerve palsy, and re-operation rate were similar between plate fixation and intramedullary nailing.

**Figure 3.**
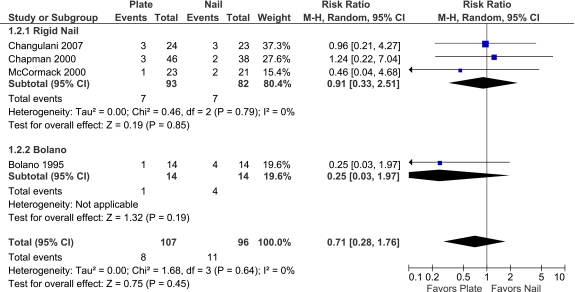
Forest plot for secondary outcome: non-union.

**Figure 4:**
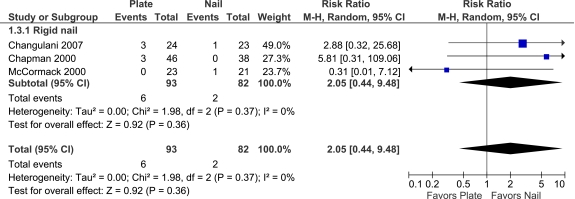
Forest plot for secondary outcome: infection

**Figure 5.**
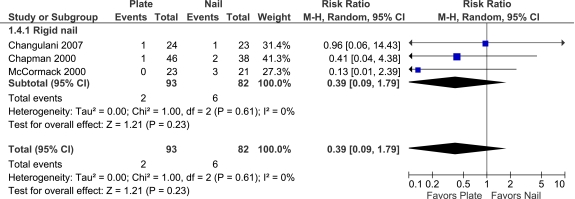
Forest plot for secondary outcome: nerve palsy.

**Figure 6.**
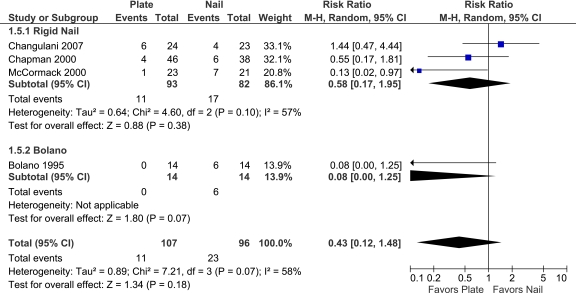
Forest plot for secondary outcome: re-operation.

Non-union rates were similar between plates and rigid nails (RR = 0.71, 95% CI: 0.28–1.76; p = 0.5). When we performed a sensitivity analysis without the Bolano abstract, these results remained inconclusive (RR = 0.91, 95% CI: 0.33–2.5; p = 0.9) (Figure 3). The infection risk associated with plates as opposed to nails was lower in plating, but was not significant (RR = 2.1, 95% CI: 0.44–9.5; p = 0.4). The Bolano abstract did not report on this outcome, so we were not able to conduct a sensitivity analysis (Figure 4). The postoperative nerve palsy with plates was lower than with nailing, but the difference was not statistically significant (RR = 0.39, 95% CI: 0.09–1.8; p = 0.2) (Figure 5). Again, a sensitivity analysis with the Bolano abstract could not be performed since there were no reported nerve palsies in this abstract. Reoperation rates were not significantly different between plate fixation and intramedullary nailing (RR = 0.43, 95% CI: 0.12–1.5; p = 0.2). The results remained inconclusive when we conducted a sensitivity analysis without the Bolano abstract (RR = 0.58, 95% CI: 0.17–2.0; p = 0.4) (Figure 6).

## Discussion

### Key findings

In this systematic review and meta-analysis, no statistically significant differences in the primary and secondary outcomes were identified following treatment of humeral shaft fractures with plates or rigid intramedullary nails. The small sample sizes in the trials that were included left the pooled estimates underpowered to allow us to make any definitive conclusions about the best treatment for humeral shaft fractures—intramedullary nails or plate fixation.

The optimal treatment of humeral shaft fractures following failed closed management is not defined. Treatment with an intramedullary nail placed through a limited exposure and also treatment with a plate placed through a more extensile exposure are both acceptable options. In 2006, Bhandari et al. published a meta-analysis on the rate of reoperation (defined as any subsequent surgery after initial surgery) and the difference between treatment with plates and intramedullary nails. They found a statistically significant difference in favor of plate fixation over intramedullary nailing regarding reoperation rate. Since then, a new randomized controlled trial published by Changulani et al. (2007) indicated that nails were superior to plates in the treatment of humeral shaft fractures. This randomized controlled trial involved 47 patients and was thus the second largest randomized controlled trial to be published on this subject. The new randomized controlled trial provided sufficient rationale for a new meta-analysis on the subject, to provide the best possible evidence regarding the most superior treatment of humeral shaft fractures.

We failed to find a statistically significant difference, but there was a strong trend favoring plates with regard to reoperation rate. Our findings are inconsistent with the previous meta-analysis (Bhandari et al. 2006). This is interesting, since only 1 study (Changulani et al. 2007) had been added to the original meta-analysis and 1 trial that described flexible nailing had been removed (Rodríquez-Merchán 1995). The primary analysis included 203 patients for comparison of nails and plates, and suggested that plates were of more benefit; it was unfortunately too underpowered, however, to allow any definite conclusions regarding the superiority of plates over rigid nails.

### Strengths and limitations

Our study had certain limitations. The first limitation was that only 4 studies could be included. Thus, the total number of patients was small. The pooled sample sizes were not large enough for the analyses to be conclusive.

One of the trials was only available as an abstract. Despite several attempts, we were unable to obtain an unpublished full text manuscript of this study. Since the abstract did provide sufficient details about complications in both groups, we preferred to conduct analyses with and without the Bolano paper (sensitivity analyses) rather than exclude it altogether. If the study had been systematically different from the others due to poor quality, for example, our sensitivity analyses would have identified a large difference in pooled treatment effects with and without the abstract data included.

A second limitation was the heterogeneity between the studies included. For the primary outcome of total complication rate, the heterogeneity was large (I^2^ = 62%) even though this outcome showed no statistically significant difference between plates and nails. The heterogeneity was probably due to conflicting data between studies.

In addition, using the CLEAR NPT checklist made it clear that there are limitations to the studies included, revealing a strong possibility of bias. This may have been due in part to the inherent difficulty of designing a randomized controlled trial with a surgical intervention in which the patient and the care providers are blinded. It was clear there was clinical bias as well, since antegrade and retrograde nailing were all combined in 1 study group. Unfortunately, it was not possible to do a subgroup analysis for this, since the articles included did not provide data in separate groups for these interventions. Funnel plots were used to assess the bias between the studies. These plots showed scattering and asymmetry, suggesting publication bias. Since only 4 studies were included, it was not possible to assess this bias further, since further tests are not powerful enough to distinguish between chance and real asymmetry of the funnel plots (Alderson and Green 2002a).

### Previous literature

This meta-analysis is a follow-up of a previously published meta-analysis by Bhandari in 2006. The authors of that article found that there was a statistically significant difference in reoperation rate and shoulder impingement and the results favored plating over nailing. We updated this article by conducting a new literature search to identify all articles on this subject with the total complication rate as the primary outcome. We identified 1 new article subsequent to Bhandari's systematic review, which we included in the meta-analysis—and enlarged our pooled patient group by adding 47 patients, bringing the total pool of patients to 203. That article reported on all our outcomes, both primary and secondary, and thus adds statistical power to our results. We deleted 1 article that reported on flexible nailing, a technique that is no longer common practice (Rodríquez-Merchán 1995). It is remarkable that although we only included 1 additional published study and deleted 1 article, we failed to identify any differences in outcomes compared to the original meta-analysis. This is likely to be due, in part, to the contradictory findings of this single additional RCT, which favored nails; this contrasts with previous RCTs on the same topic—all of which favored plate fixation.

### Implications for future research

This underpowered analysis calls strongly for additional randomized clinical trials in the future to definitively determine which treatment option is better. A methodologically sound multicenter trial involving a large patient group would solve this issue. This would have to be a randomized controlled trial with appropriate allocation of patients and blinding of care providers/examiners. We also recommend that a future study should include important patient-based outcomes since previous studies have failed to provide self-reported information from the patient's perspective. A valid and patient-important outcome (i.e. DASH score or rates of reoperation) would be helpful in this regard (Hudak et al. 1996). This study should preferably include approximately 470 patients per arm (power = 80%, alpha = 0.05, with 30% risk reduction).
